# Breed classification of Lao People's Democratic Republic (Lao PDR) and Thai native chickens using synchrotron radiation-based Fourier transform infrared spectroscopy and genotyping by sequencing

**DOI:** 10.1016/j.psj.2026.107456

**Published:** 2026-07-17

**Authors:** Phannika Khoutsavang, Rujjira Bunnom, Saknarin Pengsanthia, Pornchanok Phanhiran, Satoshi Kubota, Douangta Douangvilay, Thongnoun Phonxaiya, Viengsavanh Phimphachanhvongsod, Wittawat Molee, Kanjana Thumanu, Amonrat Molee

**Affiliations:** a School of Animal Technology and Innovation, Institute of Agricultural Technology, Suranaree University of Technology, Nakhon Ratchasima, 30000, Thailand; b Synchrotron Light Research Institute, Nakhon Ratchasima, 30000, Thailand; c National Agriculture and Forestry Research Institute (NAFRI), Vientiane, 01170, Lao People's Democratic Republic

**Keywords:** Breed classification, Genotyping-by-sequencing, Lao People's Democratic Republic (Lao PDR) native chicken, Thai native chickens, Synchrotron FTIR spectroscopy

## Abstract

Lao PDR harbors substantial genetic diversity in native chicken populations, representing an important resource for sustainable production and long-term food security. This study aimed to classify five Lao native chicken breeds—Ou, Black Bone, Horn Chou, Yolk, and Chae—and to discriminate them from a Thai native breed, Leung Hang Khao (LK), using integrative genotype-based approaches. Blood samples were collected from 50 LK and Lao native chickens (32 Ou, 10 Black Bone, 9 Horn Chou, 121 Yolk, and 41 Chae). Genomic DNA was extracted and analyzed using synchrotron radiation-based Fourier-transform infrared (SR-FTIR) spectroscopy to characterize biochemical composition, while genotyping-by-sequencing (GBS) was employed to identify genome-wide single nucleotide polymorphisms (SNPs). SR-FTIR analysis revealed highly significant differences among breeds in nucleotide-associated functional groups, including thymine, adenine, guanine, cytosine, as well as DNA backbone and deoxyribose components (P < 0.001). Multivariate analyses demonstrated that principal component analysis (PCA) of SR-FTIR spectra effectively discriminated chicken breeds, while hierarchical cluster analysis (HCA) further resolved them into two major clusters with distinct sub-clusters, reflecting variation in DNA biochemical composition. In contrast, GBS analysis identified 1484 common SNPs; however, PCA based on SNP data showed limited resolution in clearly separating breeds, despite revealing similar clustering trends. Overall, the results highlight the strong discriminatory power of SR-FTIR spectroscopy for rapid and effective classification of native chicken breeds at the molecular level, outperforming SNP-based differentiation under the current marker density. This study provides novel insights into the application of synchrotron-based spectroscopic techniques in poultry genetics and contributes valuable baseline information for the conservation and utilization of Lao native chicken genetic resources.

## Introduction

Native chickens are important in the agricultural and cultural life of Southeast Asia, serving as an essential protein source for rural people and genetic diversity (Terfa et al., 2019; Lawal and Hanotte, 2021; Wongloet et al., 2023). However, their populations are declining due to the rise of commercial breeds, disease outbreaks, and unsustainable poultry industry practices (Hoffmann et al., 2005; Larbi et al., 2013; World Bank, 2021). Specifically, Lao PDR is a country that has high agro-biodiversity as well as genetic diversity of native chickens (Prosperity, 2003; Khamhoung and Van Gansberghe, 2016). Thus, it is necessary for strategies for the sustainable conservation of the genetic diversity of native chickens.

Breed classification of native chickens is a crucial first step in sustainable conservation (Khanyile et al., 2015), as it helps in breed verification and the identification of unique genetic traits (Getu, 2014; Jeong et al., 2016; De Jesus Almeida et al., 2023). However, one of the few studies by Bouahom et al. (2007) classified Lao native chicken breeds based on phenotypes, including skin, comb, and feather color. The information is still not comprehensive, as there is currently no data to classify the breeds based on genetic information. Therefore, it should be used to classify Lao native chickens, as it can enhance the opportunity for selecting chickens to improve the breed. Understanding breed differences helps maintain desirable characteristics and increase production without losing genetic diversity. In addition to Lao native chicken breeds, the Thai native breed, Leung Hang Khao (LK), from Suranaree University of Technology was benchmarked. This breed has a well-documented genetic background and has undergone systematic selection, whereas Lao native chickens have not been subjected to such selection. Therefore, it is suitable for evaluating breed differentiation. Its inclusion helps to clarify whether the observed variation among Lao native chickens reflects true genetic divergence or shared regional characteristics.

Currently, synchrotron radiation-based Fourier transform infrared (SR-FTIR) spectroscopy is recognized as a sensitive and powerful technique for detecting subtle changes in molecular composition and vibrational characteristics. It provides detailed information on the biochemical composition of biological samples, including proteins, lipids, and nucleic acids (Yu, 2004; Miller and Dumas, 2006). SR-FTIR spectroscopy has been shown to detect structural alterations and damage in nucleic acids by analyzing changes in functional groups associated with bases and sugar–phosphate backbones (Dovbeshko et al., 2000). In addition, this technique enables the analysis of DNA within intact cells, making it particularly suitable for biological samples such as chicken blood, where nucleated erythrocytes provide direct access to genomic material (Whelan et al., 2011). Moreover, SR-FTIR offers a rapid and non-destructive approach for analyzing DNA structure and composition compared to conventional methods (Thumanu et al., 2009). It has also been successfully applied for molecular fingerprinting and classification in various biological systems, including plant varieties and microorganisms (Dziuba et al., 2007; Song et al., 2014; Phansak et al., 2021; Lee et al., 2019; Kassem et al., 2023). Although SR-FTIR spectroscopy has been used to classify breeds based on genomic DNA, it has not yet been applied to the classification of animals. Based on the successes of previous studies, we hypothesize that SR-FTIR can classify native chicken breeds at the genomic DNA level, as this technique detects both the vibrations of chemical bonds and the total biochemical composition of genomic DNA (Froehlich et al., 2011; Zelig et al., 2011; Pakbin et al., 2022). Therefore, the present study examines this technique for detecting the transformation, composition, and structure of DNA to classify breeds of native chickens. Our study provides a new perspective and understanding of rapid classification based on differences in the molecular composition and structure of DNA.

Genotyping by sequencing (GBS) is an uncomplicated technique and high multiplex capabilities that are based on next-generation sequencing (NGS) of subsets of fragments created by specific restriction enzymes (Kim et al., 2016; Wang et al., 2020). This approach enables genotyping of thousands of single nucleotide polymorphisms (SNPs) across the genome, making it highly suitable for breed classification (Gurgul et al., 2019; Pootakham et al., 2023). It has been successfully used to classify breeds of sheep, cattle, pigs, chickens, horses, and goose (De Donato et al., 2013; Wang et al., 2015; Pértille et al., 2016; Palaiokostas et al., 2018; Grzegorczyk et al., 2021). In this study, we employed GBS to classify native chicken breeds because it provides a high-resolution assessment of genetic variation, which is essential for distinguishing closely related breeds and understanding their genetic structure. Thus, this study would like to classify native chicken breeds in Laos using SR-FTIR spectroscopy and GBS.

In the present study, we aimed to classify Lao native chicken breeds based on their biochemical composition at the DNA level using Synchrotron Radiation Fourier Transform Infrared (SR-FTIR) spectroscopy. This approach enables the detection of subtle changes in the sugar–phosphate backbone of DNA, which may reflect differences in DNA structural stability and genetic robustness. The results from this study provided an extensive database on native chicken breed classification for use in confirming the Lao native breed and determining genetic and management improvements in the production and conservation of native chickens.

## Materials and methods

### Ethics statement

The study was conducted following all procedures that were approved by the Ethics Committee on Animal Use of the Suranaree University of Technology, Nakhon Ratchasima, Thailand; document ID: COA. SUT-IACUC-003/2024.

### Sample collection

**Fig. 1:**
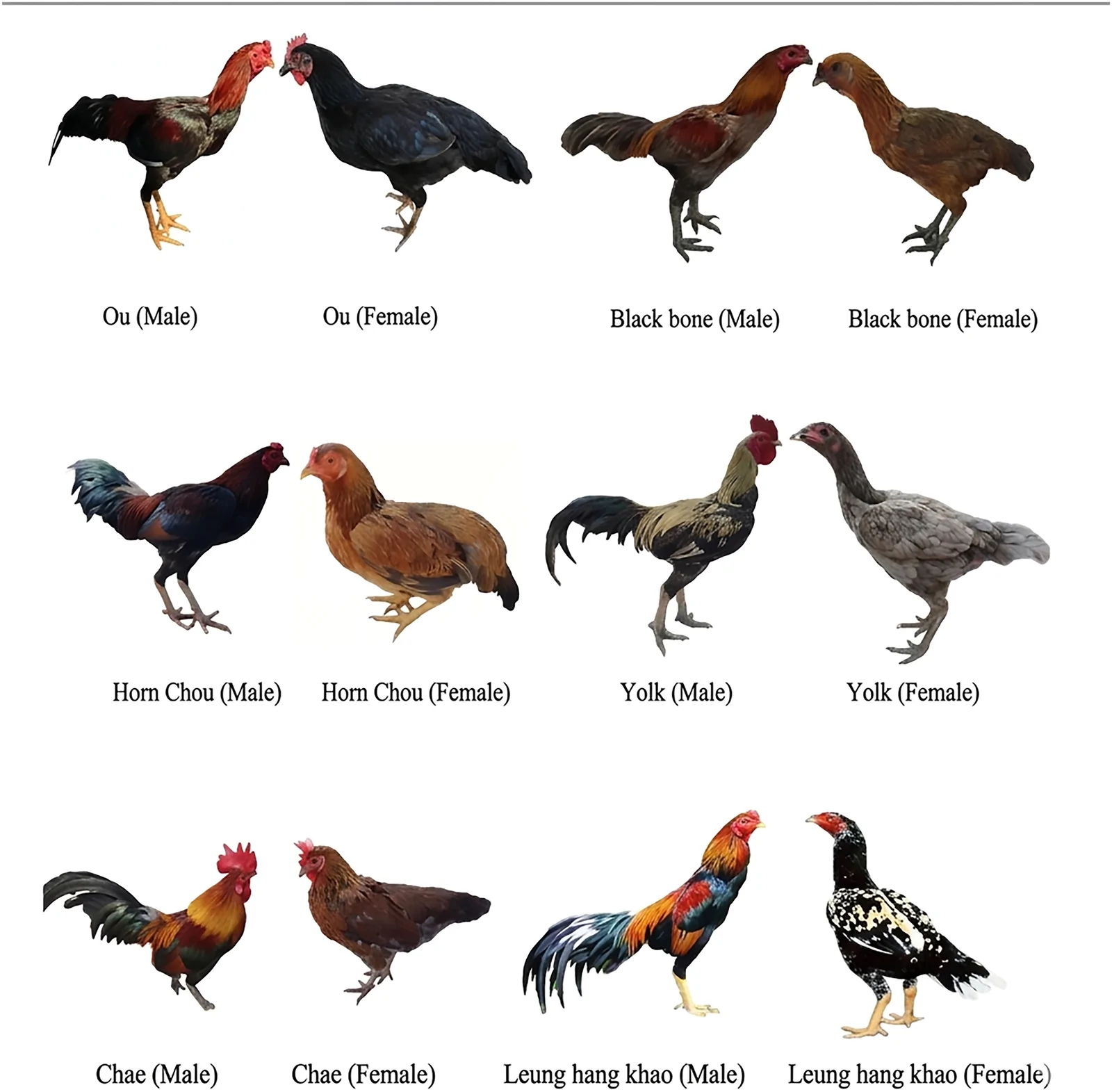
Phenotypic characteristics of six native chicken breeds, including five Lao native chickens (Ou, Black bone, Horn Chou, Yolk, and Chae) and Thai native chicken (Leung Hang Khao).

Native chickens in three provinces of Lao People’s Democratic Republic (Lao PDR), including Phongsaly, Loungprabang, and Savannakhet were classified the breeds by their phenotypes (color, leg color, and comb type) following the method of FAO (2019) and Bouahom et al. (2007). The phenotypic characteristics of all chicken breeds are shown in Fig. 1.

Blood samples were aseptically collected from the wing vein. In total, 213 samples were obtained from five Lao native chicken breeds (Ou, n = 32; Black bone, n = 10; Horn Chou, n = 9; Yolk, n = 121; Chae, n = 41), and 50 Thai native chicken (Leung Hang Khao) was used as a reference population that was supplied by the Center of Excellence on Technology and Innovation for Korat Chicken Business Development, Suranaree University of Technology, Nakhon Ratchasima, Thailand. All of the blood samples were stored at −20°C until they were extracted genomic DNA

### Genomic DNA extraction

The genomic DNA was extracted from the 263 blood samples using a GF-1 Blood Genomic DNA kit (Vivantis Technologies, Malaysia) according to the instructions provided by the manufacturer. Genomic DNA quality and quantity were determined via Nanodrop 2000 spectrophotometer (Thermo Fisher, CA, USA) at wavelengths of 280, 260, and 230 nm, and 1% agarose gel electrophoresis. The final concentration of DNA was adjusted to 100−200 ng/µl and temporarily stored at −20 °C.

### Sample preparation and SR-FTIR spectroscopy measurement of genomic DNA

SR-FTIR spectroscopy was performed to analyze 263 genomic DNA samples. The purified genomic DNA (2 µl) was loaded onto the BaF2 window, then dried and prewarmed to 37°C by a desiccator (Thermo Fisher) for 40–50 min. SR-FTIR spectra were measured at beamline BL4.1 IR Spectroscopy and Imaging at the Synchrotron Light Institute (SLRI, Nakhon Ratchasima, Thailand). The samples were analyzed the chemical structure change and DNA composition by an SR-FTIR spectrometer on a Bruker FTIR spectrometer (Vertex70, Bruker Optics, Ettlingen, Germany) coupled to a Bruker Hyperion 2000-IR Microscope (Bruker Optik GmbH, Ettlingen, Germany) with a 36x objective, connected to a mercury cadmium telluride (MCT) detector cooled with liquid nitrogen over a 4000–800 cm^-1^ measurement range. The SR-FTIR spectra were obtained in the transmission mode at a resolution of 4 cm^-1^ with 64 scans at a 10 × 10 µm aperture. The wavenumber range of 1800–900 cm^-1^ was used, and the classification of the band assignment was shown in Table 1. SR-FTIR spectral acquisition and analysis were performed using the software OPUS 7.5 (Bruker Optics Ltd, Ettlingen, Germany). The integral areas were determined using second-derivative processing at the spectral regions from 1800 to 900 cm^-1^, including thymine (1700−1664 cm^-1^), adenine (1640−1575 cm^-1^), guanine (1540−1520 cm^-1^), cytosine (1492−1300 cm^-1^), DNA backbone (1232−1060 cm^-1^), and deoxyribose (1053−900 cm^-1^).

**Table 1:** The band assignments of chemical structures and composition of genomic DNA.

Wavenumber (cm^-1^)	Assignment	Molecular vibration of functional group	Refs.
1704, 1700–1664	Thymine	C=O stretching C2=O2 C4=O4	Banyay et al. (2003) Froehlich et al. (2011) Serec et al. (2016)
1600–1575 1660–1640 1639–1600; 1690, 1608; 1609; 1610	Adenine	C=N stretching C8=N7 PO_2_^-^ asymmetric	Marty et al. (2009) Froehlich et al. (2011) Serec et al. (2016)
1540–1527 1710–1716 1509; 1425; 1698	Guanine	C =O stretching In-plane vibration	Marty et al. (2009) Froehlich et al. (2011)
1529; 1492–1300 1294; 1491	Cytosine	In-plane vibration	Froehlich et al. (2011)
1238; 1240 1232–1223 1190; 1080; 1060 1058	DNA backbone	V_as_ PO_2_^-^ O–P–O antisymmetric stretching C—O vibration	Sofińska et al. (2020) Pakbin et al. (2022) Zelig et al. (2011)
1053–900 966; 967; 968 970	Deoxyribose	C-O deoxyribose stretching	Froehlich et al. (2011) Zelig et al. (2011) Serec et al. (2016) Duan et al. (2023)

### Genotyping by sequencing (GBS) library preparation and sequencing

Genotyping by sequencing analysis was performed using 54 genomic DNA samples from 6 breeds (9 samples per breed). GBS library preparation, sequencing, and analysis were executed by Novogene Biotechnology Company (Novogene, Beijing, China) in cooperation with WARD MEDIC LTD (Bangkok, Thailand) according to the manufacturer’s instructions. Briefly, each genomic DNA sample was digested by the *EcoRI* and *MseI* restriction enzymes. The acquired DNA fragments were ligated with two barcoded adapters, each possessing a compatible sticky end with the primary digestion enzyme as well as the Illumina P5 or P7 universal sequence. All of the samples were pooled and size-selected for the necessary fragments (approximately 360 bp) to complete constructing the library following several rounds of PCR amplification. Pair-end sequencing was executed on the Illumina Novaseq 6000 instrument (Illumina, San Diego, CA, USA) platform, with a read length of 150 bp at each end.

### Single nucleotide polymorphism (SNP) detection and annotation

The original image data produced by Illumina sequencers were transformed into sequence reads through base calling with the Illumina pipeline CASAVA v1.8.2 software and subjected to the procedure of quality control to remove unusable reads. Sequencing reads were then aligned to the chicken reference genome (RefSeq assembly accession: GCA_000002315.5) using BWA (Li and Durbin, 2009) version 0.7.8-r455 with default parameters. The raw SNP sets were called by samtools (Li and Durbin, 2009) version 0.1.19-44428 cd with the parameters as ‘-q 1 -C 50 -m 2 -F 0.002 -d 1000′. The mapping quality > 20 and the depth of the variate position > 4 were applied. Following filtering of minor allele frequency (MAF) 0.01, Max_missing no missing (100%), Min Dp 10, Max Dp 200, Min GQ 30. The annotation and effect prediction of SNPs were also performed by the open-source program SnpEff version 5.1d (http://snpeff.sourceforge.net/).

### Data analysis

#### Significant difference analysis of SR-FTIR spectroscopy

The integral area of the biochemical composition of DNA was examined by Tukey's multiple tests, and the significant difference between each breed was computed. Statistical significance was determined at *P* < 0.05 using SPSS software (Version 25.0; SPSS Inc., Chicago, IL, USA).

#### Principal component analysis (PCA) of DNA biochemical composition from SR-FTIR spectroscopy, and genotyping by sequencing (GBS)

PCA was performed to identify the chemical composition of genomic DNA in the spectral range from 1800−900 cm^-1^. All spectral data were preprocessed using the Unscrambler® X multivariate data analysis software (version 10.1, Camo Analytics, Oslo, Norway). The preprocessing involved second derivative transformation with the Savitzky-Golay algorithm (13 smoothing points) and normalization using extended multiplicative signal correction. For the PCA, 300 spectra per breed were averaged. The correlation loading plot was used to illustrate the associations between variables, and all data were evaluated using a standard deviation (SD) weighting process.

PCA was also performed on imputed genotype data for breed differentiation analysis, using the glPCA function in R software (version 4.3.1) to analyze genlight objects.

#### Hierarchical cluster analysis (HCA) dendrograms of DNA biochemical composition from SR-FTIR spectroscopy, and hierarchical cluster analysis (HCA) dendrograms of genotyping by sequencing (GBS)

Cluster analysis was performed on the integral area of the biochemical composition data using the SR-FTIR spectrum data derived from genomic DNA. The spectra were analyzed using hierarchical cluster analysis (HCA) through Unscrambler® X Multivariate Data Analysis software (version 10.1, Camo Analytics, Oslo, Norway). The HCA employed the Savitzky-Golay algorithm for second derivative transformations with 13 smoothing points, which were normalized across the fingerprint region of 1800–900 cm^-1^, to identify the most similar IR spectra based on interspectral distance. For genotype data obtained from GBS, HCA was performed directly on the genotype data. For both datasets, Ward’s method based on squared Euclidean distance was applied, and cluster robustness was assessed using 10,000 bootstrap resampling iterations in PAST version 4.04 software.

## Results

### Biochemical composition of DNA in Lao and Thai native chickens by SR-FTIR spectra

**Fig. 2:**
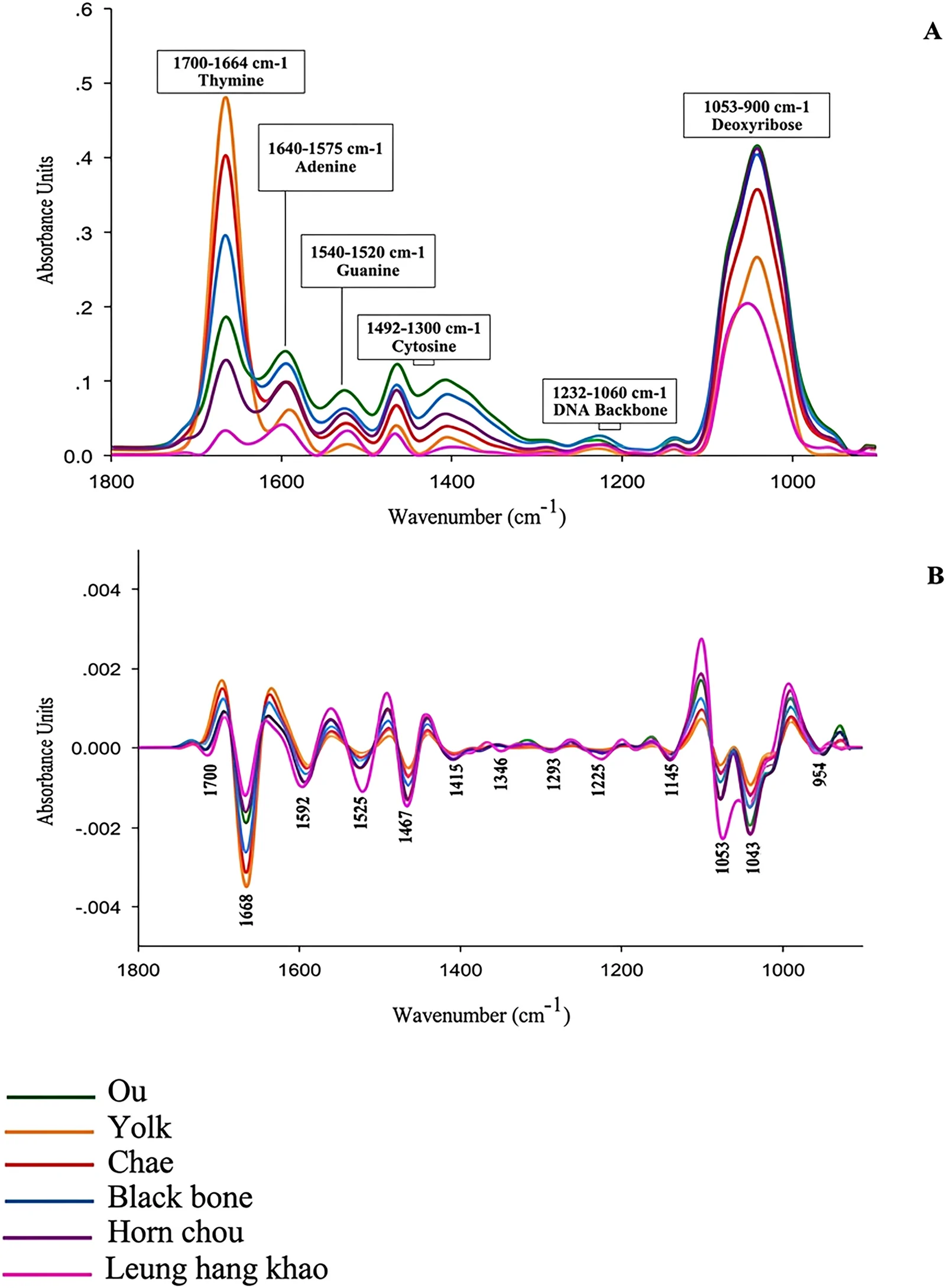
Average SR-FTIR spectra of original spectra (A) and second derivative spectra (B) from Genomic DNA of native chickens (six breeds). Spectra were collected in the spectral range of 1800 to 900 cm^−1^, resolution 4 cm^−1^ based on 300 spectra per breeds.

The average original and second derivative spectra in the fingerprint region of the wavenumber at 1800−900 cm^-1^ were detected from 263 blood samples of Lao and Thai native chicken breeds. The SR-FTIR spectra and wavenumber assignment for the classification of native chicken breeds are shown in Fig. 2A and 2B, respectively. The significant difference (*P* *<* *0.05*) in the average second derivative spectra of genomic DNA from six native chicken breeds was detected in the ranges of 1700−1664 cm^-1^ representing thymine, 1640−1575 cm^-1^ representing adenine, 1540−1520 cm^-1^ representing guanine, 1492−1300 cm^-1^ representing Cytosine, 1232−1060 cm^-1^ representing backbone, and 1053−900 cm^-1^ representing deoxyribose.

The integral areas under the peaks were obtained for the thymine, adenine, guanine, cytosine, backbone, and deoxyribose regions. The integration percentages for each chemical composition are listed in Table 2. The results showed that DNA chemical compositions including thymine, adenine, guanine, cytosine, backbone, and deoxyribose were highly significantly different among chicken breeds (*P* < 0.001). These results suggest that the SR-FTIR analysis of DNA from different Lao and Thai native chicken breeds revealed distinct biochemical compositions across six regions that affect DNA characteristics. Additionally, PCA score plot and correlation loading of the chemical composition to make the data more informative.

**Table 2:** The integral area of average spectra of the DNA chemical composition of Yolk, Chae, Black Bone, Ou, Horn Chou and Leung Hang Khao using synchrotron radiation-based Fourier transform infrared (SR-FTIR) spectroscopy.

Chemical composition of DNA (wavenumber)	Integral area (A.U)						SEM	*P*-value
	Ou	Yolk	Chae	Black Bone	Horn Chou	Leung Hang Khao		
Thymine (1700-1664 cm^-1^)	6.240^c^	13.593^a^	10.844^b^	10.979^b^	5.519^c^	3.951^d^	0.449	<0.001
Adenine (1640-1575 cm^-1^)	5.005^c^	12.431^a^	9.545^b^	7.821^b^	4.865^c^	4.535^c^	0.589	<0.001
Guanine (1540-1520 cm^-1^)	3.646^ab^	1.820^c^	2.262^c^	3.264^b^	3.400^ab^	4.080^a^	0.261	<0.001
Cytosine (1492-1300 cm^-1^)	11.125^a^	4.839^c^	7.367^b^	8.350^b^	10.840^a^	12.299^a^	0.504	<0.001
DNA Backbone (1232-1060 cm^-1^)	3.832^ab^	1.540^b^	1.214^b^	1.420^b^	2.166 ^ab^	6.381^a^	1.412	<0.001
Deoxyribose (1053-900 cm^-1^)	19.340^a^	12.493^b^	13.083^b^	12.633^b^	18.928^a^	19.393^a^	1.287	<0.001

A.U: Absorbance Units.

^a-d^ Mean values with different superscripts in the same row indicate significantly different at *P*-value < 0.05.

### Principal component analysis (PCA) of DNA chemical composition in Lao and Thai native chickens based on SR-FTIR spectra

**Fig. 3:**
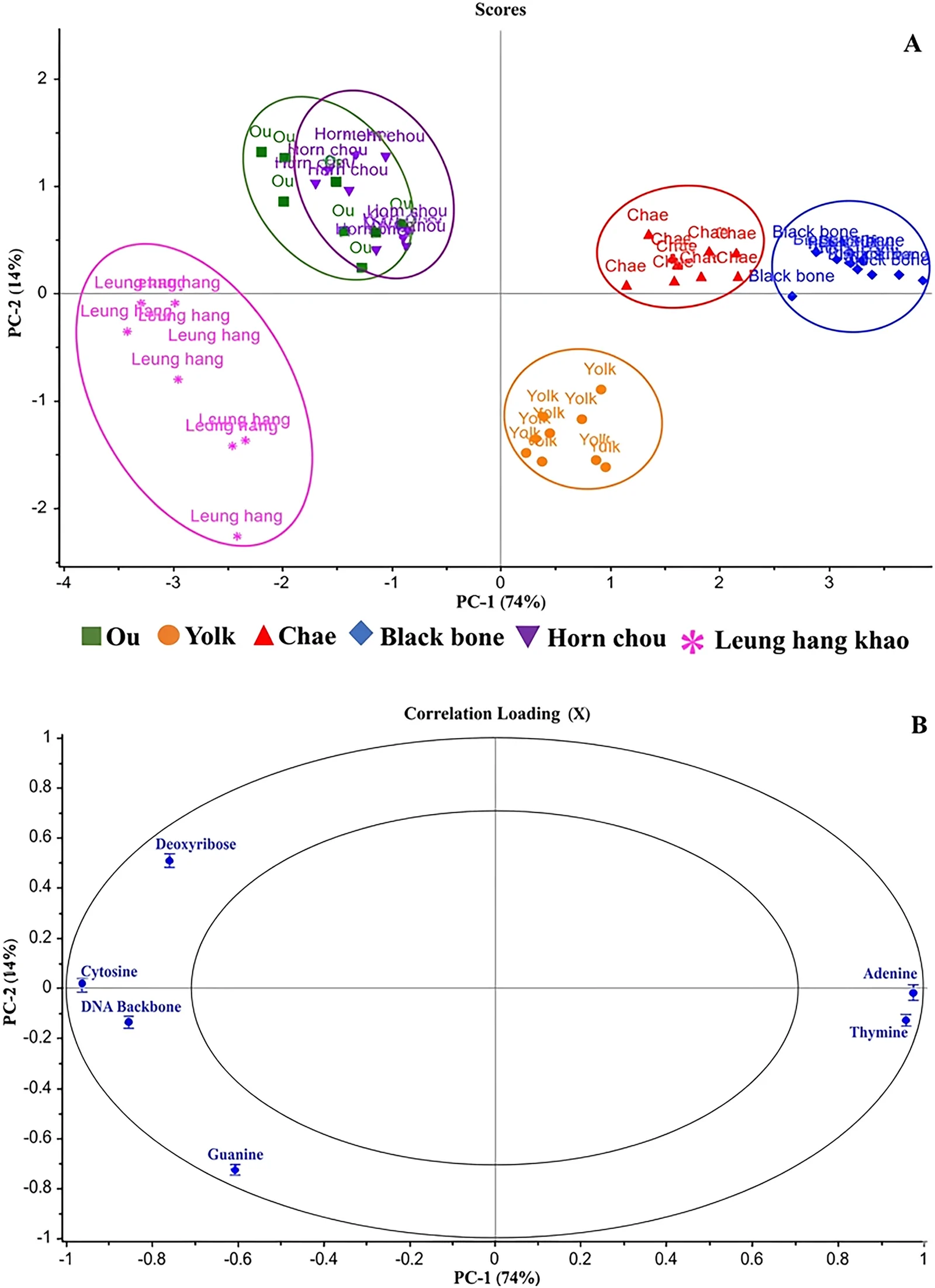
Principal component analysis (PCA) score plot (A) PC-1 versus PC-2 at 88% total variance from Genomic DNA of native chickens (six breeds), and correlation loading plot (B) detail of the effects of genomic DNA chemical composition of native chickens (six breeds). Spectra were collected 300 spectra per breeds using second derivative processing at spectral regions from 1800 to 900 cm^-1^.

To differentiate the SR-FTIR spectra of DNA from different breeds of native chickens, the PCA and HCA assays were performed. The PCA score plot and correlation loading plot reveal the relationship between SR-FTIR spectra and breeds (Fig. 3A and B). The SR-FTIR spectra revealed that the chemical compositions were separated into six groups following to the breed classification. PCA score plot and correlation loading plot was performed on the integral area from second derivative spectra compared between five Lao native chicken breeds, (Ou, Black bone, Horn Chou, Yolk, and Chae), together with LK, explained by PC-1 (74% variance) and PC-2 (14% variance) of the total variance of 88%. Positive score plot from Chae, Black bone and Yolk were associated with positive loading plot on PC-1 from thymine and adenine. In contrast, negative loading plot on PC-1 from guanine, cytosine, DNA backbone was corresponded to the Negative score plot from LK. These results indicate that different native chicken breeds possess distinct biochemical compositions in their DNA and can be classified based on their semi-quantitative DNA chemical compositions, highlighting the unique biochemical characteristics of each breed.

### Hierarchical cluster analysis (HCA) dendrograms of Lao and Thai native chickens based on SR-FTIR spectra

**Fig. 4:**
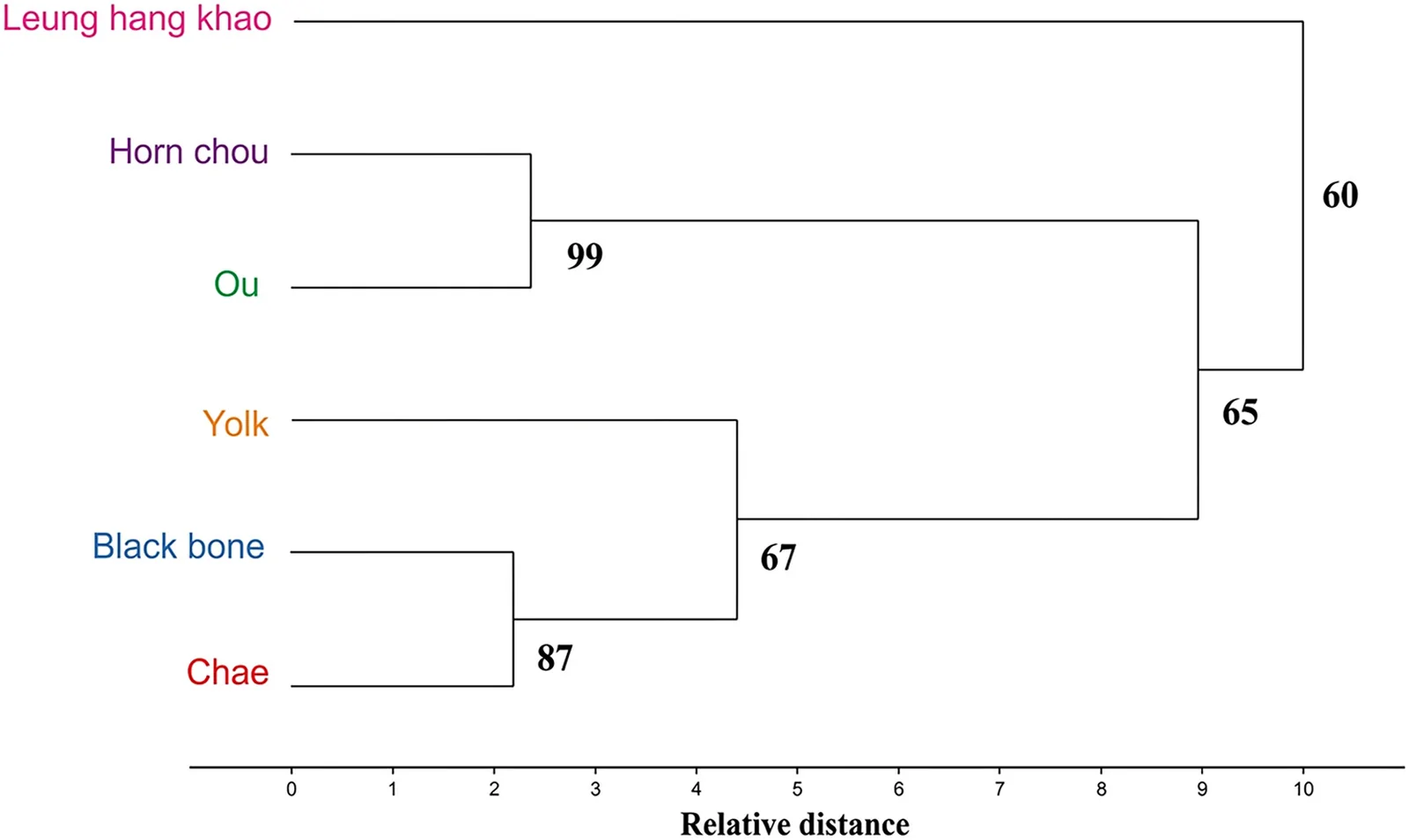
Hierarchical cluster analysis (HCA) dendrogram from PCA, the average spectra of genomic DNA from six breeds, with a bootstrap of 10,000 obtained by Ward's method by squared Euclidean distance.

The hierarchical cluster analysis (HCA) dendrogram was largely consistent with the principal component analysis (PCA) results derived from the SR-FTIR spectra, indicating concordance between the two multivariate approaches. As shown in Fig. 4, the six chicken breeds were grouped into two principal clusters. Cluster 1 comprised LK, which was separated from the Lao native breeds; however, the bootstrap support for this separation was relatively low (60%), suggesting limited robustness of this distinction. Cluster 2 included all Lao native chicken breeds and was further subdivided into two sub-clusters with moderate support (bootstrap value = 65%). Sub-cluster 1 consisted of Ou and Horn Chou, which were closely associated and strongly supported (bootstrap value = 99%), indicating a stable and reliable grouping. Sub-cluster 2 comprises Yolk, Chae, and Black Bone. Within this group, Chae and Black Bone showed relatively strong clustering support (87%), while the association of Yolk with this sub-cluster was supported at a moderate level (67%), indicating a less stable relationship.

Overall, the results suggest a degree of differentiation between the Thai native breed (LK) and the Lao native chickens; however, the low bootstrap support indicates that this separation should be interpreted with caution. In contrast, the strong clustering observed between Ou and Horn Chou, as well as between Chae and Black Bone, reflects more robust genetic or biochemical similarity. These findings highlight the presence of both distinct and shared characteristics among Lao native chicken breeds, while emphasizing the need for cautious interpretation of less-supported groupings.

### DNA sequencing data and principal component analysis (PCA) of genotyping by sequencing (GBS) in Lao and Thai native chickens

A total of 52.184 Gb of raw data was generated from 54 samples in this sequencing run, resulting in 52.164 Gb of clean data after filtering out low-quality data. The raw data production for each sample ranged from 334.455 to 1528.76 million reads, indicating a sufficient amount of data, with Q20 and Q30 scores reaching 92.65% and 82.4%, respectively, the sequencing quality meets the proper analysis requirements. The GC content varied between 39.55% and 42.23%. On average, the ratio of high-quality reads mapped to the reference genome was 99.50%, and an average of 647,463 SNPs were detected (Supplementary Table S1). After filtering the genotype data, 1484 SNPs were identified.

**Fig. 5:**
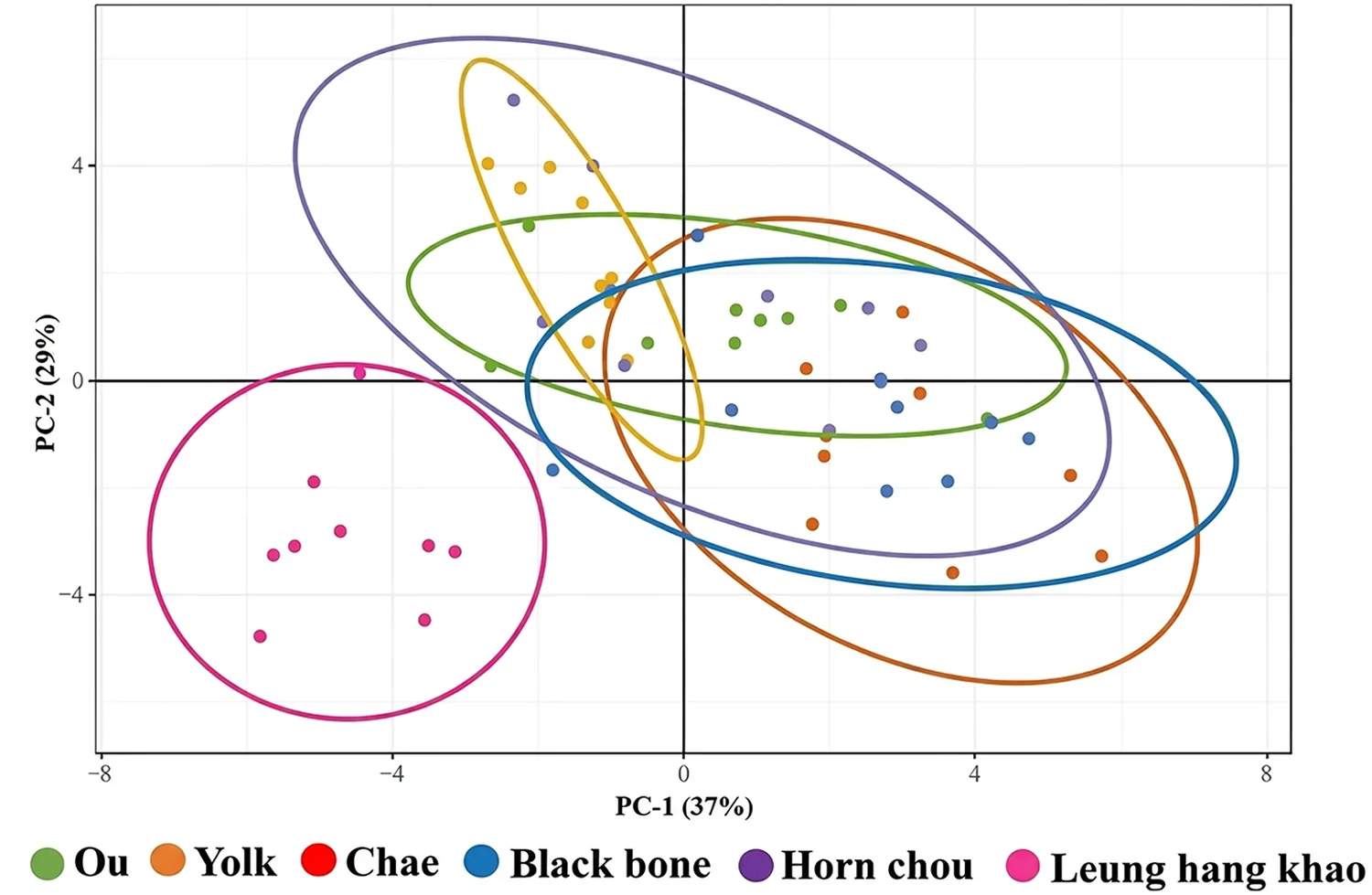
Principal component analysis (PCA) based on GBS data for native chickens (six breeds).

The PCA approach was initially used to analyze the genetic differentiation of the native chicken breed, which described about 66% of their genetic variation. This analysis revealed a distinctly distant genetic profile for the LK chicken breed, which was separated from the five Lao native breeds. In contrast, the Yolk, Ou, Horn Chou, Chae, and Black Bone breeds were not clearly distinguished, as shown in Fig. 5. These findings reveal no distinct separation between the breeds of Lao native chickens; however, they contribute to our understanding of the genetic structure of native chickens.

### Hierarchical cluster analysis (HCA) dendrograms of Lao and Thai native chickens based on genotyping by sequencing (GBS)

**Fig. 6:**
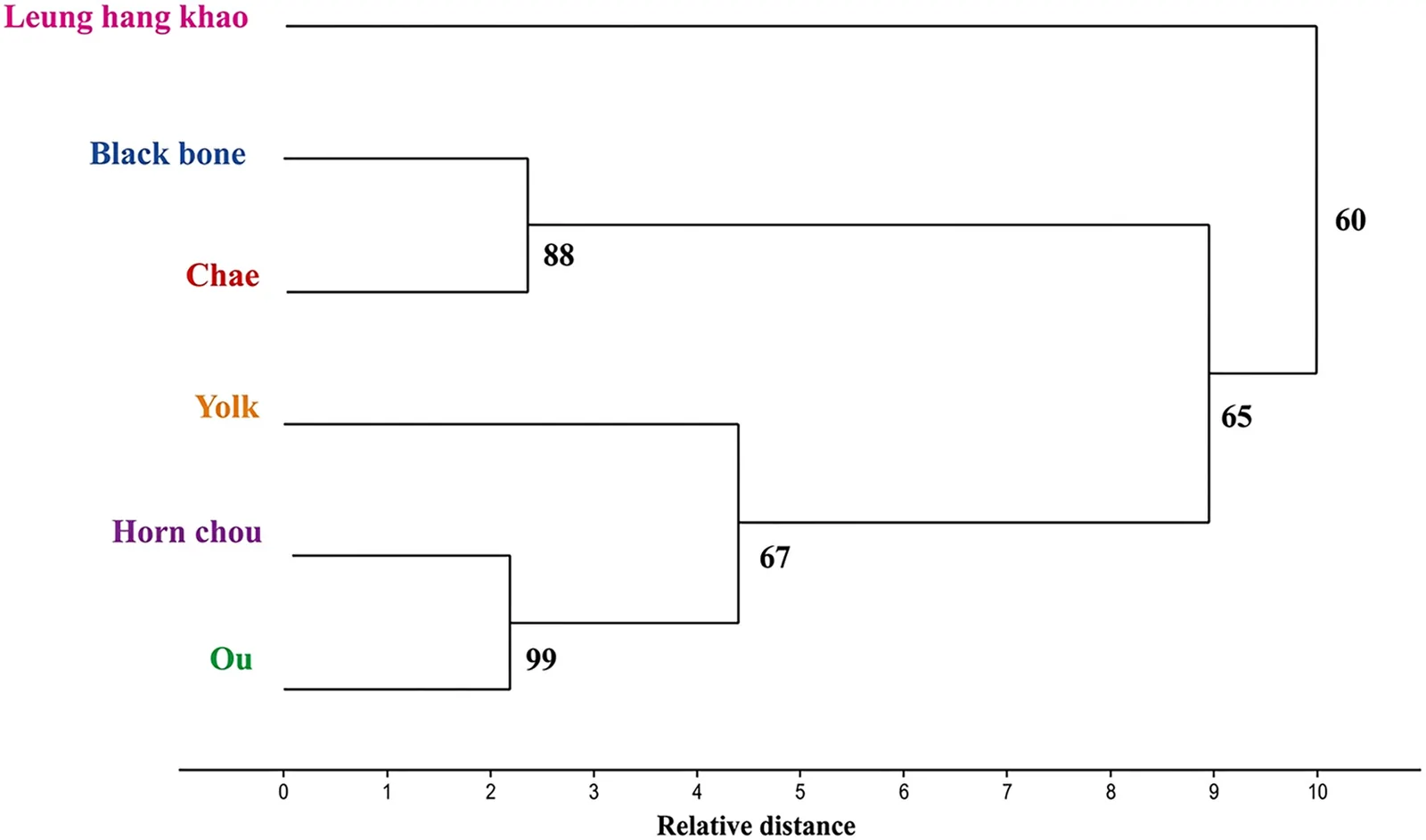
Hierarchical cluster analysis (HCA) dendrogram of native chickens (six breeds), with a bootstrap of 10,000 obtained using Ward's method by squared Euclidean distance.

The HCA dendrogram revealed two primary clusters (Fig. 6). Cluster 1 comprised Leung Hang Khao (LK), which was separated from the other breeds; however, the bootstrap support for this separation was relatively low (60%), indicating limited robustness of this distinction. Cluster 2 included all Lao native chicken breeds and was further divided into two sub-clusters with moderate support (bootstrap value = 65%). Within Cluster 2, sub-cluster 1 consisted of Chae and Black Bone, which were grouped together with relatively strong support (bootstrap value = 88%), suggesting a stable association. Sub-cluster 2 was further subdivided, with Yolk forming a separate branch supported by a moderate bootstrap value (67%), while Ou and Horn Chou were closely associated with very strong support (bootstrap value = 99%), indicating a highly reliable grouping. Overall, these results suggest a degree of differentiation between LK and the Lao native breeds; however, the low bootstrap support warrants cautious interpretation. Among the Lao native chickens, the strong clustering of Ou and Horn Chou, as well as the relatively well-supported grouping of Chae and Black Bone, reflects more consistent similarity patterns, whereas the placement of Yolk appears less stable.

## Discussion

The results of SR-FTIR align with the initial hypothesis that it can effectively classify chicken breeds by analyzing the nucleotide base functional groups in genomic. The results revealed an overlap between the Ou and Horn Chou breeds, despite their distinct phenotypic characteristics. The overlap observed between the Ou and Horn Chou breeds in the SR-FTIR analysis can be explained by similarities in their biochemical composition at the molecular level. SR-FTIR spectroscopy detects vibrational modes of functional groups in biomolecules, particularly those related to nucleic acids, such as phosphate groups, nitrogenous bases, and deoxyribose, which collectively reflect the structural and compositional characteristics of nucleic acids. The similarity in spectral profiles between the two breeds suggests that their DNA biochemical properties are highly comparable. This may be attributed to underlying genetic similarity. The overall biochemical composition of DNA—such as base composition and molecular conformation—may not differ sufficiently to generate distinct spectral separation. Previous studies have shown that FTIR spectroscopy is sensitive to the biochemical composition of biological samples and that samples with similar molecular characteristics often exhibit overlapping spectra (Movasaghi et al., 2008; Baker et al., 2014). In addition, infrared spectral features of nucleic acids are influenced by base composition and structural conformation (Naumann, 2000). For this reason, we could explain the overlap between the breeds.

The separation of LK from Lao native breeds indicates the differences in the molecular composition of DNA and the effects of past selection among breeds. These genetic differences may have resulted from environmental adaptations specific to the regions. In addition, previous studies have demonstrated that Thai native chickens, including Leung Hang Khao, possess distinct genetic characteristics and population structure compared with other indigenous and commercial chicken populations (Mekchay et al., 2014). These differences may have been shaped by long-term breeding practices and selection pressures, leading to the observed genetic distinction between Thai and Lao native chickens. Importantly, our findings highlight that the biochemical composition of genomic DNA, as revealed by SR-FTIR spectra, has the potential to be used for chicken breed classification.

The inconsistency between the SR-FTIR and GBS results likely reflects the fundamentally different biological information captured by these two approaches (Movasaghi et al., 2008; Elshire et al., 2011). In the present study, SR-FTIR-based PCA provided clearer discrimination among Lao native chicken breeds, whereas PCA based on GBS-derived SNPs showed considerable overlap among Yolk, Ou, Horn Chou, Chae, and Black Bone. This suggests that the genomic markers obtained through GBS were insufficient to resolve breed differentiation in this population.

One possible explanation is the reduced-representation nature of GBS. Because this method targets only a subset of genomic regions adjacent to restriction enzyme cut sites (Elshire et al., 2011; Li and Wang, 2017), it may fail to detect informative variants distributed across other functional regions of the genome. In closely related native chicken populations, where divergence may be modest and controlled by many loci of small effect (Muir et al., 2008; Rubin et al., 2010; Groenen et al., 2011), limited genomic coverage can substantially reduce the power to detect population structure. Therefore, the weak separation observed in the GBS-based PCA should not be interpreted as evidence of an absence of genetic differentiation, but rather as an indication that the marker set generated in this study may not have been sufficiently informative for breed discrimination. In contrast, SR-FTIR captures the overall biochemical profile of genomic DNA (Banyay et al., 2003; Movasaghi et al., 2008) and may therefore reflect cumulative molecular differences among breeds. Such differences may arise not only from sequence variation, but also from broader changes in DNA composition and molecular organization (Kazarian and Chan, 2006; Baker et al., 2014). This may explain why SR-FTIR showed a greater ability to separate breeds than GBS under the present experimental conditions. However, this interpretation should also be made with caution, because FTIR-based discrimination reflects biochemical distinctiveness rather than direct evidence of genetic distance in the population genetic sense.

Taken together, these findings support the hypothesis that breed differentiation among Lao native chickens may be influenced by dispersed genomic variation and higher-order molecular features that are not adequately captured by reduced-representation SNP genotyping. Under this hypothesis, SR-FTIR may be more sensitive to integrated molecular differences, whereas GBS requires greater marker density to achieve comparable discriminatory power. Future studies should test this hypothesis using whole-genome sequencing or high-density SNP panels, combined with larger and more balanced sample sizes across breeds. Integrating these data with transcriptomic, epigenetic, or metabolomic analyses would further improve understanding of how genomic variation is translated into breed-specific characteristics.

## Conclusion

This study revealed substantial diversity among Lao native chicken breeds (Yolk, Chae, Black Bone, Ou, and Horn Chou) when benchmarked against the Thai native Leung Hang Khao. SR-FTIR spectroscopy enabled classification of Lao native chickens into distinct groups broadly consistent with external characteristics, although complete separation was not achieved in all cases. In contrast, GBS-based SNP analysis showed limited resolution among Lao breeds, despite clearly distinguishing Leung Hang Khao from the Lao populations.

Importantly, this study is the first to integrate biochemical compound data derived from genomic DNA with SNP data from GBS for the classification of Lao native chickens. These findings highlight the potential of molecular compound-based approaches as complementary tools for breed discrimination and represent an important step toward the application of molecular-level data for native chicken characterization in Lao PDR within an international research context.

## Author contributions

Phannika Khoutsavang: Writing - original draft, Writing - review & editing, Visualization,Methodology, Data curation, Conceptualization. Rujjira Bunnom: Writing - review & editing, Methodology, Investigation. Saknarin Pengsanthia: Writing - review & editing, Visualization, Software. Pornchanok Phanhiran: Writing - review & editing, Formal analysis, Software. Satoshi Kubota: Writing - review & editing, Data curation, Methodology. Douangta Douangvilay: Writing - review & editing, Data curation, Resources. Thongnoun Phonxaiya: Writing - review & editing, Data curation, Resources. Viengsavanh Phimphachanhvongsod: Writing - review & editing, Data curation, Resources, Supervision. Wittawat Molee: Writing - review & editing, Methodology, Validation. Kanjana Thumanu: Writing - review & editing, Supervision, Investigation. Amonrat Molee: Writing - review & editing, Supervision, Resources, Project administration, Funding acquisition, Conceptualization.

## Disclosures

The authors declare that they have no known competing financial interests or personal relationships that could have appeared to influence the work reported in this paper.

## Footnotes

Supplementary material associated with this article can be found, in the online version, at doi:10.1016/j.psj.2026.107456.
