# Supplementary material for: Breed classification of Lao People's Democratic Republic (Lao PDR) and Thai native chickens using synchrotron radiation-based Fourier transform infrared spectroscopy and genotyping by sequencing
Source: Poult Sci. 2026 Jul 17;105(11):107456. doi: 10.1016/j.psj.2026.107456 (PMC13589135; doi:10.1016/j.psj.2026.107456)
Supplement: Supplementary file 1 [file mmc1.docx]

**Table S1.** Summary of data filtration and sequence quality for DNA genotyping by sequence of native chickens with different breeds. (A (Ou), B (Yolk), C (Chae), D (Black Bone), E (Horn Chou), and F (Leung Hang Khao)).

| Sample | Raw base (bp) | Clean base (bp) | Q20 (%) | Q30 (%) | GC  content (%) | Total reads | Mapped reads | Mapping rate (%) | Average depth (×) | | Number of SNPs |
| --- | --- | --- | --- | --- | --- | --- | --- | --- | --- | --- | --- |
| A1 | 872,462,592 | 872,107,382 | 95.47 | 87.94 | 41.44 | 6,058,500 | 6,032,411 | 99.57 | | 5.51 | 624,367 |
| A2 | 906,355,872 | 905,893,420 | 97.20 | 92.23 | 41.41 | 6,293,784 | 6,266,478 | 99.57 | | 5.92 | 662,075 |
| A3 | 885,348,864 | 884,788,550 | 97.42 | 92.72 | 41.89 | 6,147,932 | 6,121,839 | 99.58 | | 5.66 | 687,637 |
| A4 | 790,957,152 | 790,487,092 | 96.68 | 90.86 | 41.70 | 5,492,488 | 5,466,346 | 99.52 | | 5.58 | 629,239 |
| A5 | 859,760,352 | 859,351,378 | 96.63 | 90.77 | 41.17 | 5,970,234 | 5,942,817 | 99.54 | | 5.69 | 621,443 |
| A6 | 769,675,392 | 769,198,962 | 95.84 | 88.75 | 41.79 | 5,344,746 | 5,318,732 | 99.51 | | 5.25 | 636,460 |
| A7 | 766,754,496 | 766,423,314 | 95.15 | 87.25 | 41.53 | 5,324,486 | 5,301,144 | 99.56 | | 5.18 | 627,707 |
| A8 | 755,722,656 | 755,520,384 | 96.67 | 90.81 | 40.64 | 5,247,784 | 5,225,012 | 99.57 | | 4.95 | 582,817 |
| A9 | 747,954,432 | 747,609,518 | 95.63 | 88.28 | 41.62 | 5,193,908 | 5,171,687 | 99.57 | | 5.30 | 597,604 |
| B1 | 427,029,408 | 426,867,796 | 92.65 | 82.40 | 42.23 | 2,965,404 | 2,951,769 | 99.54 | | 3.89 | 481,460 |
| B2 | 754,839,648 | 754,563,244 | 96.75 | 91.02 | 41.23 | 5,241,650 | 5,217,356 | 99.54 | | 5.36 | 596,602 |
| B3 | 801,517,536 | 801,057,768 | 96.86 | 91.34 | 41.63 | 5,565,820 | 5,540,731 | 99.55 | | 5.68 | 621,879 |
| B4 | 334,455,264 | 334,400,548 | 96.35 | 90.26 | 39.55 | 2,322,464 | 2,309,911 | 99.46 | | 3.13 | 374,446 |
| B5 | 694,104,768 | 693,828,104 | 96.61 | 90.74 | 40.81 | 4,819,880 | 4,796,266 | 99.51 | | 4.43 | 576,116 |
| B6 | 748,800,000 | 748,492,512 | 96.30 | 89.97 | 41.17 | 5,199,772 | 5,173,273 | 99.49 | | 5.06 | 596,717 |
| B7 | 687,147,264 | 686,961,238 | 96.72 | 90.99 | 40.45 | 4771600 | 4,750,079 | 99.55 | | 5.08 | 537,839 |
| B8 | 735,476,832 | 735,276,260 | 96.59 | 90.64 | 40.27 | 5,107,168 | 5,0835,18 | 99.54 | | 4.34 | 576,176 |
| B9 | 764,546,688 | 764,147,902 | 96.59 | 90.59 | 41.58 | 5,309,042 | 5,285,833 | 99.56 | | 5.16 | 623,509 |
| C1 | 592,389,216 | 592,207,756 | 95.52 | 88.03 | 40.73 | 4,113,612 | 4,095,082 | 99.55 | | 3.79 | 558,831 |
| C2 | 663,519,744 | 663,283,668 | 97.29 | 92.33 | 40.97 | 4,607,496 | 4,588,582 | 99.59 | | 4.91 | 579,617 |
| C3 | 603,450,144 | 603,259,086 | 96.66 | 90.85 | 40.59 | 4,190,384 | 4,170,812 | 99.53 | | 4.06 | 549,854 |
| C4 | 758,193,408 | 757,779,522 | 97.14 | 92.01 | 41.83 | 5,264,872 | 5,243,095 | 99.59 | | 5.35 | 636,511 |
| C5 | 756,349,632 | 755,929,820 | 96.90 | 91.41 | 41.38 | 5,252,106 | 5,228,298 | 99.55 | | 5.40 | 576,579 |
| C6 | 534,479,328 | 534,364,790 | 96.25 | 89.90 | 39.74 | 3,711,452 | 3,694,580 | 99.55 | | 4.05 | 475,018 |
| C7 | 1,069,988,544 | 1,069,787,984 | 95.94 | 89.48 | 40.28 | 7,430,422 | 7,392,832 | 99.49 | | 5.99 | 655,029 |
| C8 | 1,116,770,400 | 1,116,277,026 | 95.86 | 89.19 | 41.83 | 7,755,298 | 7,713,079 | 99.46 | | 6.50 | 752,800 |
| C9 | 1,119,346,272 | 1,119,020,218 | 95.76 | 88.96 | 40.95 | 7,773,188 | 7,734,485 | 99.50 | | 5.91 | 720,816 |

**Table S1.** Summary of data filtration and sequence quality for DNA genotyping by sequence of native chickens with different breeds. (A (Ou), B (Yolk), C (Chae), D (Black Bone), E (Horn Chou), and F (Leung Hang Khao)) (continue).

| Sample | Raw base (bp) | Clean base (bp) | Q20 (%) | Q30 (%) | GC  content (%) | Total reads | Mapped reads | Mapping rate (%) | Average depth (×) | Number of SNPs |
| --- | --- | --- | --- | --- | --- | --- | --- | --- | --- | --- |
| D1 | 1,300,948,704 | 1,300,480,144 | 96.38 | 90.69 | 41.49 | 9,034,320 | 8,987,780 | 99.48 | 7.26 | 746,213 |
| D2 | 1,268,095,392 | 1,267,545,034 | 96.81 | 91.78 | 41.32 | 8,806,152 | 8,756,082 | 99.43 | 7.47 | 699,036 |
| D3 | 1,528,759,584 | 1,528,187,910 | 96.53 | 90.98 | 41.22 | 10,616,314 | 10,562,146 | 99.49 | 7.44 | 790,236 |
| D4 | 1,219,186,656 | 1,218,821,302 | 96.37 | 90.68 | 41.07 | 8,466,516 | 8,418,311 | 99.43 | 7.21 | 686,000 |
| D5 | 1,211,894,208 | 1,211,466,972 | 97.00 | 92.21 | 41.27 | 8,415,878 | 8,374,483 | 99.51 | 6.55 | 723,287 |
| D6 | 1,260,264,384 | 1,259,732,502 | 96.67 | 91.38 | 41.70 | 8,751,792 | 8,707,782 | 99.50 | 7.35 | 741,211 |
| D7 | 1,109,038,176 | 1,108,724,602 | 95.51 | 88.34 | 41.27 | 7,701,600 | 7,662,179 | 99.49 | 6.36 | 700,805 |
| D8 | 1,455,035,904 | 1,454,394,452 | 96.01 | 89.72 | 41.73 | 10,104,354 | 10,049,644 | 99.46 | 8.41 | 744,135 |
| D9 | 1,127,090,016 | 1,126,634,642 | 96.33 | 90.58 | 41.44 | 7,826,960 | 7,783,262 | 99.44 | 6.71 | 680,159 |
| E1 | 1,028,447,712 | 1,028,106,314 | 95.34 | 88.04 | 40.96 | 7,141,982 | 7,102,932 | 99.45 | 6.23 | 659,440 |
| E2 | 1,306,521,792 | 1,305,947,850 | 96.87 | 91.97 | 41.31 | 9,073,020 | 9,025,613 | 99.48 | 7.39 | 740,355 |
| E3 | 1,179,821,376 | 1,179,145,904 | 97.10 | 92.47 | 41.59 | 8,193,150 | 8,152,692 | 99.51 | 6.91 | 745,560 |
| E4 | 1,155,978,720 | 1,155,480,330 | 96.37 | 90.63 | 41.59 | 8,027,586 | 7,983,934 | 99.46 | 7.03 | 711,556 |
| E5 | 1,166,934,528 | 1,166,586,426 | 96.38 | 90.72 | 40.95 | 8,103,654 | 8,060,827 | 99.47 | 6.77 | 685,655 |
| E6 | 1,374,923,520 | 1,374,292,596 | 95.77 | 89.01 | 41.60 | 9,548,016 | 9,499,372 | 99.49 | 7.63 | 770,888 |
| E7 | 1,092,966,624 | 1,092,569,408 | 95.28 | 87.87 | 41.52 | 7,590,012 | 7,550,414 | 99.48 | 6.98 | 690,514 |
| E8 | 1,277,163,360 | 1,276,922,920 | 96.43 | 90.78 | 40.51 | 8,869,142 | 8,824,501 | 99.50 | 6.59 | 701,273 |
| E9 | 1,183,178,016 | 1,182,612,452 | 95.72 | 88.88 | 41.38 | 8,216,486 | 8,173,381 | 99.48 | 6.80 | 710,021 |
| F1 | 863,228,448 | 862,915,856 | 93.28 | 83.50 | 41.88 | 5,994,618 | 5,963,944 | 99.49 | 6.01 | 615,639 |
| F2 | 959,109,696 | 958,847,228 | 96.50 | 90.98 | 41.13 | 6,660,446 | 6,625,110 | 99.47 | 6.00 | 633,795 |
| F3 | 1,242,025,632 | 1,241,538,240 | 96.53 | 91.09 | 41.62 | 8,625,118 | 8,580,697 | 99.48 | 6.93 | 714,622 |
| F4 | 683,921,952 | 683,857,316 | 96.17 | 90.34 | 39.72 | 4,749,418 | 4,720,195 | 99.38 | 4.16 | 520,766 |
| F5 | 1,124,940,096 | 1,124,652,552 | 96.29 | 90.56 | 40.91 | 7,812,032 | 7,768,652 | 99.44 | 5.89 | 674,989 |
| F6 | 1,046,763,072 | 1,046,407,310 | 96.06 | 89.94 | 41.46 | 7,269,130 | 7,226,724 | 99.42 | 6.37 | 668,022 |
| F7 | 937,619,136 | 937,430,802 | 96.45 | 90.95 | 40.34 | 6,511,188 | 6,474,870 | 99.44 | 5.83 | 610,406 |
| F8 | 1,256,478,048 | 1,256,247,226 | 96.49 | 90.90 | 40.66 | 8,725,486 | 8,682,480 | 99.51 | 5.95 | 688,874 |
| F9 | 1,305,935,136 | 1,305,380,406 | 96.41 | 90.67 | 41.80 | 9,068,942 | 9,025,323 | 99.52 | 6.83 | 750,397 |
| Average | 966,364,181 | 965,996,554 | 96.23 | 90.10 | 41.18 | 6,710,719 | 6,707,092 | 99.50 | 5.89 | 647,463 |

Q20 and Q30 represent the percentage of bases with higher Phred quality score than 20 and 30 in total bases.

GC represents the percentage of G and C in total bases.
